# Effect of low-dose, high-frequency advanced life support training versus annual full-day training on simulation-based resuscitation performance: a randomized controlled trial

**DOI:** 10.1186/s12909-026-09717-3

**Published:** 2026-06-20

**Authors:** Lisa Moll, Reimer Riessen, Philipp Dahlmann, David Häske

**Affiliations:** 1 Department of Internal Medicine, Intensive Care Unit, University Hospital Tübingen, Tübingen, Germany; 2 Deggendorf Institute of Technology, Faculty of Applied Healthcare Sciences, Deggendorf, Germany; 3 Center for Public Health and Health Services Research, University Hospital Tübingen, Osianderstraße 5, Tübingen, 72076 Germany; 4 German Red Cross, Emergency Medical Service, Reutlingen, Germany

**Keywords:** Cardiopulmonary Resuscitation, Competence, Simulation Training, Emergency Medical Services

## Abstract

**Background:**

Skill decay in advanced life support (ALS) is well documented, yet optimal training frequency remains unclear. This trial compared low-dose, high-frequency ALS training with annual full-day training regarding simulation-based resuscitation performance after one year.

**Methods:**

In this randomized, controlled, simulation-based trial, 35 emergency medical services (EMS) professionals were allocated to either low-dose, high-frequency ALS training (intervention group, *n* = 18) or a single annual full-day ALS training (control group, *n* = 17). Performance was assessed at baseline and after 12 months using a validated 29-item rating instrument covering technical and non-technical skills (NTS), with items rated on a 5-point Likert scale (1 = poor performance, 5 = excellent performance). The primary endpoint was the overall performance score for resuscitation management, defined as the mean across all items. Secondary endpoints included domain-specific performance scores (NTS, defibrillation-related, and cardio-pulmonary resuscitation [CPR] items), calculated as the mean scores for each domain, as well as time to key interventions.

**Results:**

After 12 months, the intervention group showed significantly higher overall performance scores than the control group (4.7 ± 0.2 vs. 4.2 ± 0.3; *p* < 0.001). The largest between-group difference was observed for NTS (4.8 ± 0.2 vs. 3.9 ± 0.5; *p* < 0.001). Scores for defibrillation-related and CPR-related items were also significantly higher in the intervention group. Time to CPR initiation (13 ± 2 s vs. 18 ± 7 s; *p* = 0.015), rhythm analysis (29 ± 9 s vs. 45 ± 24 s; *p* = 0.015), supraglottic airway insertion (51 ± 13 s vs. 68 ± 30 s; *p* = 0.013), as well as hands-off time (4 ± 1 s vs. 6 ± 3 s; *p* = 0.002) were significantly shorter in the intervention group. The rating instrument demonstrated good reliability (Cronbach’s alpha 0.793; intraclass correlation coefficient 0.794, 95% confidence interval 0.718–0.854).

**Conclusion:**

Low-dose, high-frequency ALS training resulted in higher simulation-based resuscitation performance scores than annual full-day training, particularly for non-technical skills and time-critical processes.

**Trial registration:**

The study is registered in the German Register of Clinical Studies under the ID DRKS00024822.

**Supplementary Information:**

The online version contains supplementary material available at 10.1186/s12909-026-09717-3.

## Introduction

Out-of-hospital cardiac arrest is a leading cause of mortality worldwide and remains associated with low survival rates [1–3]. Emergency Medical Services (EMS) play a central role within the Chain of Survival, the sequence of time-critical interventions required to improve outcomes after cardiac arrest, including early recognition, high-quality cardiopulmonary resuscitation (CPR), rapid defibrillation, and advanced life support [4–6]. Patient survival, therefore, depends on rapid response, high-quality resuscitation, and structured post-resuscitation care. Advanced Life Support (ALS) has been shown to influence CPR quality and patient survival meaningfully [7]. Despite its clinical importance, there remains limited evidence regarding the optimal structure, frequency, and delivery format of ALS training, and current resuscitation guidelines provide only limited guidance in this regard [6, 8]. While educational approaches such as simulation-based training, video-assisted debriefing, and real-time feedback systems have demonstrated improvements in individual CPR metrics, their effects on sustained ALS performance and team processes remain insufficiently understood [9].

A key challenge in resuscitation education is the well-documented phenomenon of skill decay. Although training effects are typically measurable immediately after educational interventions, their long-term retention varies substantially depending on training format, frequency, and task complexity [10–12]. Regular, feedback-based training, often delivered in small-group simulation settings, has been associated with improved resuscitation performance and skill retention [13–15].

Continuing professional education is therefore essential not only for maintaining technical competence but also for ensuring patient safety, reinforcing adherence to guidelines, and supporting effective clinical decision-making. This is particularly relevant in resuscitation, where high workload, time pressure, and complex team interactions characterize the initial treatment phase. In addition to objective performance, subjective factors such as confidence and perceived competence may also be influenced by training. However, these effects are heterogeneous and not always aligned with actual performance [16].

Meta-analytical evidence further supports the importance of structured team-based resuscitation training, demonstrating improved survival [17], with similar findings in additional analyses [18]. However, the optimal training interval for achieving and sustaining these benefits remains unclear.

Previous studies investigating repeated short training sessions, for example, among firefighters, have shown only limited improvements in Basic Life Support (BLS) performance [19]. Whether such low-dose, high-frequency approaches are more effective in the ALS context – particularly regarding team management and process quality – has not yet been adequately addressed. Given the limited time available for training among medical, nursing, and EMS personnel, there is also a need for educational strategies that can be integrated into routine clinical practice with minimal disruption while still achieving meaningful performance improvements.

### Objectives

The objective of this trial was to compare the effect of low-dose, high-frequency ALS training (monthly short sessions) versus annual full-day training on resuscitation performance in emergency medical services, measured at 12 months as the overall performance score derived from a validated 29-item rating instrument.

## Methods

### Trial design

**Fig. 1:**
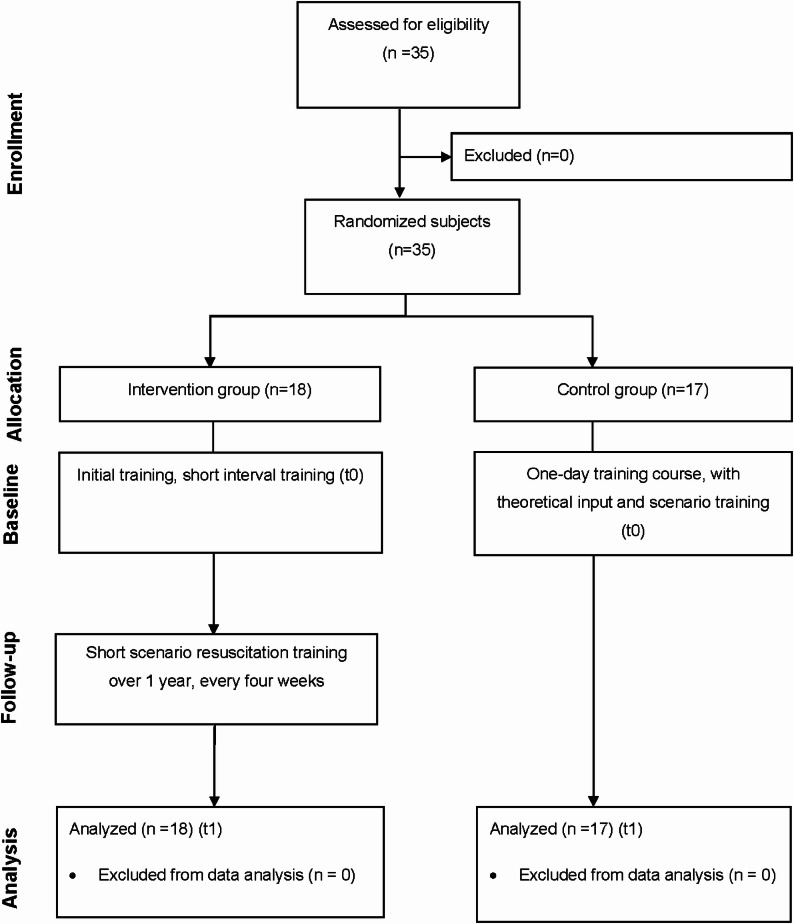
CONSORT flow diagram of participant recruitment, randomization, and follow-up. t0 = baseline assessment at study entry; t1 = follow-up assessment after 12 months

This is a non-blinded, randomized, controlled, simulation-based trial. A self-developed rating instrument was used to assess resuscitation quality during training sessions and to compare across different periods (Fig. 1). The trial was registered on March 26, 2021, in the German Registry of Clinical Trials under ID DRKS00024822 https://trialsearch.who.int/.

### Participants

Eligible participants were active, full-time EMS personnel with appropriate qualifications as paramedics (4600 h of training) or emergency medical technicians (520 h of training). No exclusion criteria were implemented. Recruitment was conducted through direct communication with EMS organizations and snowball sampling via the medical faculty. Participants received an one-time compensation at the end of the trial.

### Intervention

The intervention consisted of a low-dose, high-frequency training approach (LDHF), compared with standard annual training in the control group. Both training formats adhered to the ALS standard.

#### Intervention group

At the start of the trial, the intervention group completed a single scenario training session to establish a baseline. Participants then received theoretical instruction on current resuscitation guidelines, followed by CPR scenario training in teams of two, totaling approximately 2 h.

Subsequently, the intervention group received CPR training every 4 weeks, using a single CPR scenario in accordance with the ALS standard. The training session lasted about 10 to 15 min and concluded with brief feedback from the CPR trainers.

For the control group, an initial resuscitation scenario was likewise conducted by a team of two paramedics at the beginning of the training course to establish baseline performance.

Participants then received theoretical instruction in basic life support (BLS) and ALS according to the guidelines in effect at the time of training. Small-group ALS training scenarios were followed by subsequent feedback. After each scenario, the roles of team leader and team partner were swapped.

#### Control group

The control group received one day of training corresponding in scope to the first day of an ERC ALS course (BLS and defibrillation, theoretical introduction to the ALS algorithm, and scenario simulation training with feedback), as outlined by the European Resuscitation Council (ERC).

Both the initial theoretical lessons and the CPR scenario training were conducted and supervised by emergency medical service instructors with ERC-instructor qualifications.

The training scenarios included two shockable and two non-shockable CPR cycles, presented in an alternating order. The training focused on CPR management with high guideline adherence and process quality, particularly:

- Correct sequence and overview of the resuscitation process, focusing on resuscitation management. These include correct administration of medication (indication, timing, dosage), appropriate application of measures, exclusion of reversible causes, effective change of helpers, high adherence to guidelines, and minimal interruptions.
- Non-technical skills related to situational awareness, decision-making, leadership, teamwork, task allocation, and communication were also covered.
- Rapid recognition of the situation and taking appropriate measures.
- High-quality manual chest compressions in terms of frequency, depth, and relief without technical aids.
- Minimal interruption for rhythm analysis and correct interpretation, as well as safe and rapid defibrillation if necessary.
- Correct airway management and ventilation, including capnography.

#### Material

For simulation training, a Laerdal resuscitation manikin (MegaCode Kelly Advanced, Laerdal, Norway) was placed on floor mats. An Electrocardiogram (ECG) with an integrated defibrillator (Corpuls C3) was also used and connected to the manikin via a dedicated SimPad. Other training materials included EMS equipment, such as a breathing and circulation case/backpack containing various emergency medical supplies.

### Outcomes

#### Primary outcome

The primary endpoint was the overall performance score for resuscitation management, comprising technical, non-technical, and procedural domains, and defined as the mean score across all items. Performance was assessed at baseline and after 12 months using a validated 29-item rating instrument, with each item rated on a 5-point Likert scale (1 = poor performance, 5 = excellent performance).

#### Outcome assessment scenario

The outcome data were collected using a standardized, video-recorded simulation scenario that was identical at the start of the study (t0), during the subsequent training sessions for the intervention group, and at the follow-up assessment after 12 months (t1). Each scenario was conducted by a team of two emergency medical service personnel representing the participating emergency medical team; the focus of the evaluation was on the team leader. Roles were assigned based on the team’s own choice; however, each participating paramedic served as team leader in at least one scenario during the study. The scenario started with an unconscious adult patient found on the floor; the manikin (Laerdal MegaCode Kelly Advanced, Laerdal, Stavanger, Norway) was placed on a floor mat in a simulation room and was initialized by the instructor immediately before the team entered. The clinical course followed a standardized script comprising four resuscitation cycles, two with shockable rhythms (ventricular fibrillation / pulseless ventricular tachycardia) and two with non-shockable rhythms (asystole or pulseless electrical activity), presented in a randomized order. Rhythms and other simulator-controlled stimuli were triggered by the instructor via a dedicated SimPad in accordance with the script. Each scenario lasted approximately 10 min, followed by a brief debriefing of up to 5 min that was not part of the outcome assessment. The scenario was terminated when the predefined four-cycle sequence had been completed.

Only the participating two-person team and one instructor were present during the scenario. The instructor was responsible for starting the recording equipment, controlling the simulator, and providing standardized verbal information that could not be conveyed through the simulator itself, such as patient history and answers regarding reversible causes when explicitly requested by the team. The instructor did not intervene in resuscitation decisions and did not provide feedback during the scenario.

Scenarios were recorded with a single fixed video camera positioned at the foot of the manikin, capturing the entire team and the resuscitation area; audio was recorded simultaneously. A structured initial briefing on the assessment procedure and on the use of the manikin and equipment was provided once at study entry (t0) only; in order to avoid priming. The recorded videos were subsequently rated offline by independent assessors as described in the following sections.

#### Secondary outcome

- ▪ Duration of certain stages of the process
  - ◦ Recognizing cardiac arrest until CPR begins (seconds)
  - ◦ Recognizing cardiac arrest until the start of initial analysis (seconds)
  - ◦ Starting CPR until insertion of supraglottic airway device (SGA) (seconds)
  - ◦ Starting CPR until capnography is established (seconds)
  - ◦ Hands-off time analysis (seconds)
- ▪ Subjective safety and experience of the test subjects, as assessed using a questionnaire with rating scales

#### Development of the rating instrument and primary rating procedure

The initial literature search identified a range of validated instruments for assessing resuscitation performance and training outcomes. However, these tools consistently addressed different aspects of resuscitation and were not fully aligned with the intended scope of the present rating instrument. Some instruments primarily focus on basic life support (BLS) performance, such as the Cardiff Test of Basic Life Support [20–22]. In contrast, others emphasize broader team performance or non-technical skills, as described by Cooper and colleagues [23].

Given these conceptual differences and the lack of a comprehensive instrument tailored to this project’s specific objectives, it was decided to develop a new rating instrument rather than adapting or directly mapping existing tools. Consequently, no formal alignment with previously published rating instruments was pursued.

The present rating instrument was further refined based on exploratory video analyses of pilot CPR trainings. The identified items were subsequently reviewed by an interdisciplinary panel of six experts representing emergency medicine, intensive care medicine, medical education, emergency medical services, and methodology. Using a semi-structured consensus approach, items were iteratively discussed, modified, included, or excluded to ensure both content relevance and practical applicability.

After content revision and validation, the final rating instrument comprises four categories with 29 points reflecting technical and non-technical skills. Assessment is based on a five-point scale. The test’s reliability was confirmed by six independent experts who verified it across multiple resuscitation scenarios. The reliability test results for the six evaluators, each with 29 items, are shown in the Supplement. The consistency of the item ratings is good, with a Cronbach’s alpha of 0.793 and an intraclass correlation coefficient of 0.794 (95% confidence interval: 0.718-0.854), according to Koo and Li [24, 25].

The same panel of six independent experts who participated in the development and reliability testing of the rating instrument also performed the primary outcome rating for this trial. Each expert independently scored every video-recorded scenario using the 29-item rating instrument; ratings were not discussed between experts. For each video, the six expert ratings were aggregated by calculating the mean score per item, which was then used to derive the overall performance score and the domain-specific scores reported in the results (Table 2). The expert raters were blinded to group allocation. Discrepancies between individual expert ratings were not resolved by consensus but reflected in the aggregated mean score and in the reliability statistics (Cronbach’s alpha 0.793; ICC 0.794, 95% CI 0.718–0.854).

#### Supplementary blinded outcome assessment

To assess the robustness and objectivity of the primary outcome rating described above, an independent supplementary rating of the same video recordings was performed by a separate group of raters. Six students from the Bachelor of Science in Emergency Medical Services Education program at Technical College Deggendorf, who were not involved in the development of the rating instrument or in the primary rating, independently evaluated all 13 video scenarios using the instrument. Raters were blinded to group allocation. Disagreements between the six student raters were not resolved by consensus; instead, ratings were aggregated by calculating mean scores per video. These supplementary scores were compared with the primary outcome ratings to assess the robustness of the primary results.

#### Questionnaire

A self-developed questionnaire was used to collect demographic characteristics, prior clinical experience, and safety-related information (see Supplementary Material).

Participants were asked about their experience in resuscitation and post-resuscitation care. In addition, they rated their confidence in the use and management of technical equipment during resuscitation and provided a self-assessment of their competencies. All items were rated on a four-point Likert scale ranging from 1 (“strongly agree”) to 4 (“strongly disagree”).

### Sample size

No directly applicable literature was available for planning the sample size. As an approximation, an initial evaluation of pilot scenario-based training sessions was performed using the rating instrument, focusing on the primary endpoint, the overall performance score for resuscitation management. This evaluation yielded a standard deviation of 0.25 on the 1–5 Likert scale. Based on this estimate, a between-group difference of 20% was considered the minimum relevant effect, as smaller differences are unlikely to translate into meaningful changes in performance, even in applied training contexts.

Based on this estimate, a 20% difference between the groups was considered the minimum relevant effect, since smaller differences are unlikely to result in significant changes in performance, even in applied training contexts. In the pilot scenario-based training sessions, the application of the rating instrument yielded a standard deviation of 0.20. Thus, 17 participants per group would be required to reject the null hypothesis of equal population means with a power of 0.8. The probability of a Type I error associated with this test was set at α < 0.05.

### Randomization: sequence generation

A random allocation sequence was generated using IBM SPSS Statistics (IBM Corp., Armonk, NY, USA). Participants were assigned identification numbers in order of enrollment, and simple randomization with a 1:1 allocation ratio was applied.

### Randomization: allocation concealment mechanism

Allocation concealment was ensured by performing randomization only after all participants had been recruited and enrolled. The allocation sequence was generated by an independent investigator (DH) and was not accessible to the investigator responsible for recruitment (LM) prior to assignment.

### Randomization: implementation

Participants were recruited by LM through invitation and enrolled if they met the predefined inclusion criteria. The random allocation sequence was generated by DH. Group allocation was performed at the beginning of the training session, after all participants were enrolled.

### Data management

The video data was stored on two hard drives until the study was finished. Afterward, the completed questionnaires and consent forms were archived at the study site. In accordance with the requirements of the Medical Faculty of the University of Tübingen, the session protocols and interim results were recorded in a logbook. Only the previously agreed-upon contact details were used to organize the study.

### Statistical methods

Descriptive statistics were reported as mean ± standard deviation (SD) for normally distributed data and as median with first and third quartiles (Q1, Q3) for non-normally distributed data. Frequencies are reported in absolute and relative terms. Statistically significant values were defined as *p* < 0.05. Group differences were assessed using the χ² test for categorical variables. For continuous variables, the independent-samples t-test was used when data were approximately normally distributed; otherwise, the Mann-Whitney U test was used. In the absence of baseline imbalances and given the study’s focus on post-training performance, no adjustment was applied to pre-intervention scores. Effect sizes were calculated using Hedges’ g to account for bias arising from the small sample size. Where appropriate, confidence intervals (95%) were reported.

Intraclass correlation (ICC) with a “two-way random effects” model (3.1) was used to determine inter-rater reliability [26]. According to the literature, values ≥ 0.75 are recommended to assess inter-rater reliability. Statistical analyses were performed with SPSS Statistics 28 (IBM SPSS Statistics).

## Results

### General and demographic data of the test subjects

A total of 35 subjects participated in the study, 18 in the intervention group and 17 in the control group. Table 1 lists the general and demographic data of the test subjects.

**Table 1:** Demographic characteristics of the participants

Characteristics	Intervention group, *n* = 17	Control group, *n* = 18
Sex, % (*n*)		
Female	35.3% (6)	23.5% (4)
Age, years, mean ± SD	30 ± 11	32 ± 11
Years of professional experience, mean ± SD	7.2 ± 5.9	12.2 ± 10.6
Frequency of participation in resuscitation training, % (*n*)		
3 months ago	17.6% (3)	23.5% (4)
6 months ago	5.9% (1)	0% (0)
12 months ago	76.5% (13)	76.5% (13)
Time since last real-life resuscitation, % (*n*)		
1 month ago	23.5% (4)	41.2% (7)
3 months ago	41.2% (7)	29.4% (5)
6 months ago	23.5% (4)	17.6% (3)
12 months ago	11.8% (2)	11.8% (2)

*SD* standard deviation

Both groups were comparable at the start of the study and had similar starting conditions.

### Video analysis

In comparison with the initial measurement (t0), the measurement following one year (t1) exhibited an enhancement of the overall mean performance score of 27.0% and 10.5%, respectively, in both the intervention group (3.7 ± 0.7 to 4.7 ± 0.2) and the control group (3.8 ± 0.3 to 4.2 ± 0.3) (Table 2). The largest increase in the intervention group was observed in the non-technical skills, with a 37.1% rise. The most prominent increase in the control group was observed in the defibrillation-related mean score, which rose by 15.4%.

**Table 2:** The following table presents the results of the control and intervention groups, both before and after the training intervention. In addition to the initial task, the four resuscitation cycles are presented in alternating order. Each resuscitation cycle encompassed CPR with chest compressions and ventilation (CPR-related group), electrocardiogram (ECG) rhythm analysis, ECG interpretation, and, if indicated, defibrillation (defibrillation-related group). The non-technical skills were also summarized once more as the NTS-related group, so that the groups NTS, “Defibrillation-related,” “CPR-related,” and “Overall” are available. SD=Standard deviation, CPR= cardiopulmonary resuscitation. Details of the items are provided in the Supplement

Items	Pre evaluation				Post evaluation			
	Intervention group Mean ± SD, *n* = 18	Control group Mean ± SD, *n* = 17	*p* value	Effect size Hedges’ g (95% CI)	Intervention group Mean ± SD, *n* = 18	Control group Mean ± SD, *n* = 17	*p* value	Effect size Hedges’ g (95% CI)
Overall mean score	3.7 ± 0.7	3.8 ± 0.3	0.735	0.11 (-0.54 to 0.76)	4.7 ± 0.2	4.2 ± 0.3	< 0.001	-2.13 (-2.95 to -1.28)
Non-technical skills (NTS) mean score	3.5 ± 0.7	3.6 ± 0.3	0.398	0.28 (-0.37 to 0.93)	4.8 ± 0.2	3.9 ± 0.5	< 0.001	-2.46 (-3.34 to -1.56)
Defibrillation-related mean score	3.8 ± 0.7	3.9 ± 0.4	0.410	0.28 (-0.38 to 0.92)	4.8 ± 0.4	4.5 ± 0.3	0.035	-0.74 (-1.42 to -0.05)
CPR-related mean score	3.4 ± 0.5	3.6 ± 0.4	0.391	0.29 (-0.37 to 0.94)	4.0 ± 0.2	3.7 ± 0.3	< 0.001	-1.34 (-2.07 to -0.60)

For the primary outcome, the intervention group achieved a significantly higher overall mean score than the control group at 12 months (4.7 ± 0.2 vs. 4.2 ± 0.3; *p* < 0.001), corresponding to a 11.9% relative between-group difference. Domain-specific scores were also higher in the intervention group, with the largest between-group difference observed for non-technical skills (NTS) (4.8 ± 0.2 vs. 3.9 ± 0.5; *p* < 0.001; 23.1% relative difference).

### Supplementary blinded outcome assessment

The supplementary blinded outcome assessment also showed a significantly higher mean score in the intervention group than in the control group (3.9 ± 0.7 vs. 3.3 ± 0.6; *p* < 0.001), although the absolute scores were slightly lower than in the primary assessment.

**Fig. 2:**
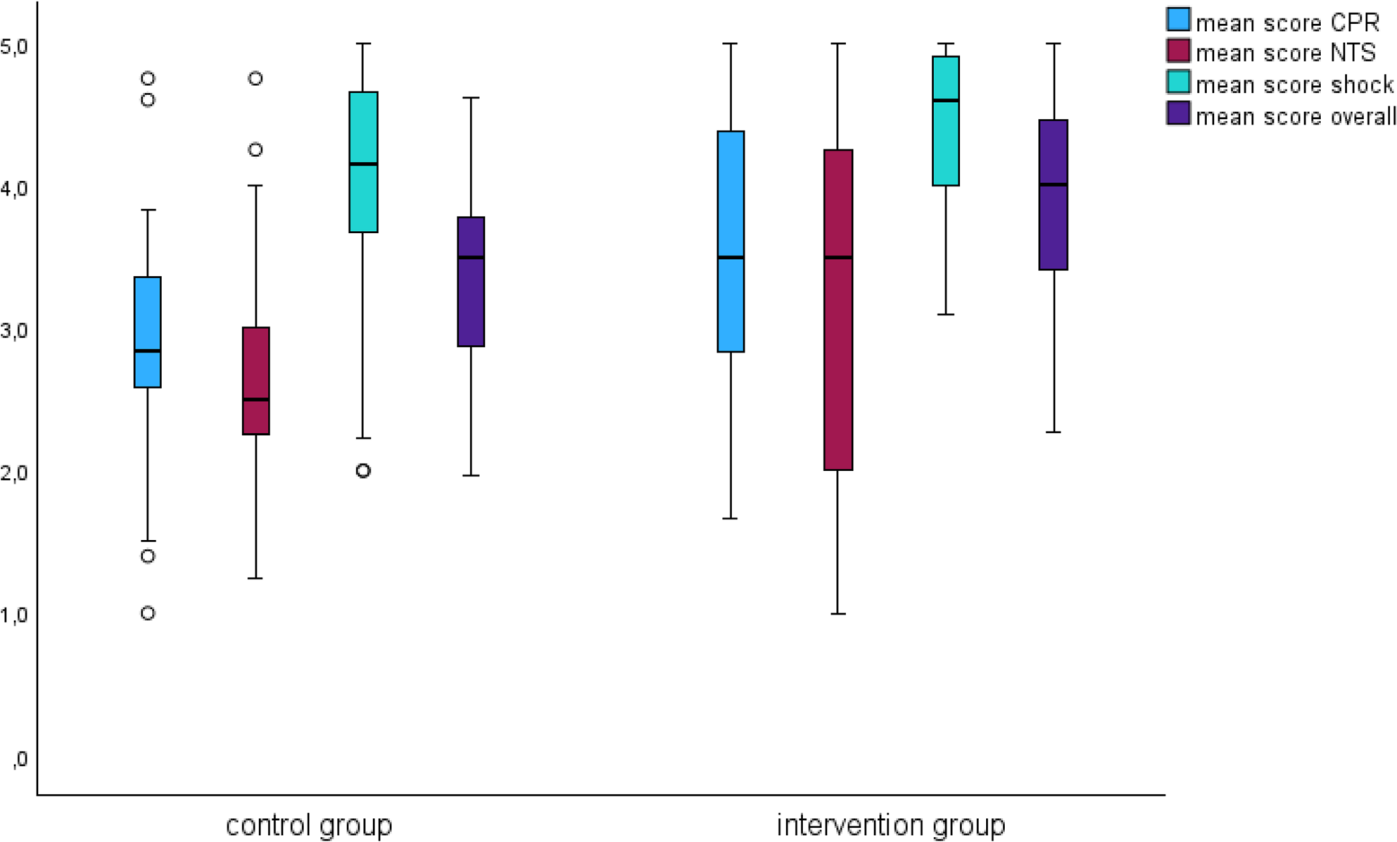
Supplementary blinded outcome assessment. CPR=Cardio-pulmonary resuscitation. NTS = Non-technical skills

The intervention group also showed higher mean scores than the control group across domains: NTS 3.3 ± 1.3 vs. 2.6 ± 0.8 (*p* = 0.006); defibrillation-related 4.4 ± 0.6 vs. 4.0 ± 0.9 (*p* = 0.020); and CPR-related 3.6 ± 0.9 vs. 2.9 ± 0.8 (*p* < 0.001) (Fig. 2).

### Quantitative analysis

The analysis of time, or the duration until measures were implemented, except for the establishment of capnography, demonstrated significant differences across the board. The interval between the onset of cardiac arrest and the initiation of treatment, or the hands-off time, was reduced in all areas within the intervention group (Table 3).

**Table 3:** Time differences between the two groups

	Intervention group Mean ± SD	Control group Mean ± SD	*p*-value
Detection of cardiac arrest until the start of CPR. Seconds	13 ± 2	18 ± 7	0.015
Detection of cardiac arrest until the start of initial analysis. Seconds	29 ± 9	45 ± 24	0.015
Time from start of CPR to insertion of SGA. Seconds	51 ± 13	68 ± 30	0.013
Time from start of CPR to application of capnography. Seconds	82 ± 16	91 ± 32	0.443
Hands-off time during ECG analysis. Seconds	4 ± 1	6 ± 3	0.002

### Questionnaire

Table 4 presents the questionnaire results on subjective safety before and after the trial in the intervention and control groups. No statistically significant differences were observed between groups.

**Table 4:** Evaluation of the questionnaire on subjective safety and experience based on a rating scale from 1 (“strongly agree”) to 4 (“strongly disagree”)

	PRE			POST		
	Intervention group Median [Q1, Q3]	Control group Median [Q1, Q3]	*p*-value	Intervention group Median [Q1, Q3]	Control group Median [Q1, Q3]	*p*-value
I feel confident in guiding my team during CPR	2 [2–2]	2 [2–2]	0.634	1 [1–2]	1 [1–2]	0.511
Do you feel adequately trained in the procedure and algorithm for CPR?	2 [1–2]	2 [1–2]	0.599	1 [1–1]	1 [1–2]	0.687
Do you feel experienced enough to maintain/guide a CPR cycle in accordance with guidelines?	2 [1–2]	2 [2–2]	0.861	1 [1–2]	1 [1–2]	0.511
Do you feel confident using a defibrillator during CPR?	1 [1–2]	2 [1–2]	1	1 [1–2]	1 [1–1]	0.511
Would you be confident in assessing the various ECG rhythms?	2 [1–2]	2 [1–2]	0.953	2 [1–2]	1 [1–2]	0.511
Would you feel confident in correctly treating the different ECG rhythms?	2 [1–2]	2 [1–2]	0.683	1 [1–2]	1 [1–2]	0.979
Do you feel adequately trained in administering medications during resuscitation?	1 [1–2]	1 [1–2]	0.786	1 [1–1]	1 [1–1]	0.762
Are you adequately trained in administering medications during resuscitation?	1 [1–2]	1 [1–2]	0.736	1 [1–1]	1 [1–1]	0.336
Are you familiar with all the potentially reversible causes of cardiac arrest?	1 [1–2]	2 [1–2]	0.669	1 [1–1]	1 [1–1]	0.762
Can you recognize ROSC during resuscitation using specific parameters?	2 [1–2]	1 [1–2]	0.683	1 [1–2]	1 [1–1]	0.511
Are you familiar with the ROSC algorithm?	2 [1–2]	2 [1–2]	0.339	1 [1–2]	1 [1–1]	0.65

## Discussion

### Principal findings

In this randomized, controlled simulation-based trial, low-dose, high-frequency ALS training resulted in significantly higher overall resuscitation performance scores at 12 months than a single annual full-day training session. The most pronounced increase was observed in NTS, accompanied by significant reductions in time to critical interventions, including earlier CPR initiation, faster rhythm analysis, and reduced hands-off time.

### Skill decay and training frequency

Skill decay in resuscitation is well documented. Procedural and cognitive competencies deteriorate within months after training, particularly when exposure to real-life cardiac arrest is limited [6, 8]. Meta-analytical findings, as well as insights from staff surveys on broader research into skill retention, confirm that infrequent training is associated with a significant decline in performance [10, 27].

Educational recommendations from the ERC emphasize the importance of regular refreshers and feedback-based training rather than reliance on infrequent intensive courses [28, 29]. The present trial extends this concept by demonstrating sustained benefits of low-dose, high-frequency ALS training over one year.

While previous investigations in BLS settings reported only modest improvements with short-cycle training [19], our results indicate a more pronounced effect in the ALS context, particularly in domains requiring cognitive integration and team coordination. This is also consistent with ILCOR’s systematic review of spaced versus massed learning, which supports distributed training as the more effective format for resuscitation skill retention [29].

An interesting parallel can be drawn to a recent clinical cohort study on in-hospital cardiac arrest by Wittig et al., which analyzed 157 resuscitation attempts across four Danish hospitals and differentiated between team leaders with recent simulation training (< 6 months) and those with substantial clinical experience (≥ 4 years) [30]. Recent simulation training was associated with significantly shorter longest chest compression pauses and a higher chest compression fraction. In contrast, years of experience as a team leader were not associated with any of the evaluated CPR quality metrics. These observations closely mirror the pattern observed in our trial, in which the control group reported greater professional experience (12.2 vs. 7.2 years) but achieved lower performance scores after 12 months than the short-interval training group. Taken together, these findings suggest that recent, deliberate practice—rather than accumulated clinical exposure alone—is the more decisive determinant of resuscitation performance, supporting the rationale for structured low-dose, high-frequency training formats.

### Beyond compression depth: why management and process quality matter

A substantial proportion of earlier CPR research focused on technically measurable parameters such as compression depth (mm), compression rate, ventilation volume (ml), and hands-off time [31, 32]. These metrics are essential determinants of coronary and cerebral perfusion and are strongly associated with outcome [33].

Modern defibrillator and monitor systems routinely capture these variables and provide real-time feedback, as recommended by the ERC Guidelines [8]. In many EMS systems, real-time audiovisual feedback on compression quality has become standard practice, and post-event debriefing based on device-recorded data is technically feasible.

However, such systems do not assess the broader management of cardiac arrest. They do not evaluate leadership, decision-making, prioritization, medication timing, structured search for reversible causes, team communication, or situational awareness. These aspects constitute the organizational and cognitive framework within which technical CPR is delivered.

The present study deliberately focused on these management and team processes. The marked increase in NTS observed in the intervention group underscores the relevance of training formats that repeatedly expose teams to structured decision-making and leadership under simulated stress.

Importantly, team-based CPR training has been associated with improved survival and neurological outcome in meta-analyses [17, 18]. Although our study did not evaluate patient outcomes, the relevant increase in process metrics and team performance supports the plausibility of downstream clinical relevance.

### Subjective confidence and safety

Both groups demonstrated increased self-reported confidence following the intervention. However, no statistically significant differences in subjective confidence were observed before or after the intervention. Notably, the control group reported slightly higher post-intervention confidence levels than the intervention group.

Several explanations may account for this finding. First, participation in the training itself may have generated heightened short-term confidence independent of the specific instructional format. In this context, a Hawthorne effect—where participants modify their responses due to awareness of being studied—cannot be ruled out [34]. Additionally, unrealistic expectations regarding the training effect may have influenced self-assessment, potentially leading to an overestimation of perceived competence.

Although differences in age and years of professional experience were not statistically significant, the control group had a higher mean number of years in service (12.2 vs. 7.2) and was slightly older. Greater clinical experience may contribute to higher self-perceived confidence, irrespective of actual performance. This experience-related effect could partially explain the higher subjective confidence ratings observed in the control group.

### Clinical relevance versus statistical significance

The intervention group demonstrated an 11.9% higher overall mean score than the control group after 1 year. While statistically robust (*p* < 0.001), the clinical relevance of this difference warrants careful interpretation. It is important to note that the performance score used here does not reflect actual clinical impact. It is a composite measure of adherence to guideline-based processes in a simulated setting. It cannot be directly translated into the probability of return of spontaneous circulation, survival to discharge, or a favorable neurological outcome.

Simulation-based performance cannot be directly equated with improved patient survival. Nevertheless, several findings support potential clinical importance:

- Reduced hands-off time during rhythm analysis
- Earlier initiation of CPR
- Improved structured management of reversible causes
- Substantial gains in non-technical skills

Even modest reductions in no-flow time may influence myocardial and cerebral perfusion during cardiac arrest [6]. Given the exponential relationship between time to intervention and survival, incremental process improvements may carry disproportionate clinical weight [35].

We deliberately chose a composite checklist score rather than a single outcome-linked metric, such as the longest chest compression pause, which has been shown to correlate with survival and neurological recovery. The rationale was threefold. First, the primary research question concerned the overall quality of resuscitation management, which encompasses not only mechanical CPR parameters but also team coordination, structured assessment of reversible causes, and non-technical skills — dimensions that a single chest compression metric cannot capture. Second, none of the mechanical parameters (e.g., compression depth, frequency, and exact duration of pauses) from the video recordings used in this study were applied to the trial because the focus was on process organization and management, as mentioned. Third, a composite score more accurately reflects the educational objective of the training intervention, which aimed to improve the entire resuscitation process rather than individual skills.

### Feedback, motivation, and integration into daily practice

Time pressure in EMS and hospital settings limits the availability of extended training sessions. Short, scenario-based sessions lasting approximately 10 to 15 min may represent a feasible alternative that integrates into the routine workflow.

Regular micro-scenarios with structured feedback may also address an often-overlooked dimension: positive performance reinforcement. In everyday practice, high-quality performance frequently remains unnoticed unless errors occur. Feedback-based training may therefore strengthen self-efficacy and perceived safety, which have been shown to improve after structured emergency training interventions [10, 11].

Small-group formats and repeated exposure have demonstrated superior educational effectiveness compared with passive or large-group instruction [13]. Our findings align with this educational paradigm.

From an educational perspective, the superiority of the short-interval training format may be explained by constructive alignment [36]. Learning objectives (guideline-adherent ALS management, including non-technical skills), instructional activities (repeated, scenario-based team simulations with structured feedback), and assessment (process-oriented rating instrument) were closely aligned. This alignment likely fostered active knowledge construction and directed attention toward management quality and team processes, thereby supporting sustained improvements beyond isolated technical CPR metrics.

### Future perspectives: AI-supported supervision and cognitive load

It should be emphasized that the present study did not employ artificial intelligence (AI) in either the training intervention or the assessment of performance. Both groups received conventional, instructor-led simulation training, and the checklist-based evaluation of video recordings was conducted manually by trained human raters. We address AI here in a forward-looking sense, as the limitations identified in our study point directly to areas where AI-based tools could complement future training and supervision concepts. While modern defibrillators provide detailed mechanical CPR feedback, they do not assess cognitive and organizational aspects of resuscitation. Emerging technologies may help close this gap. Randomized studies of supervised AI-based decision support in emergency scenarios showed higher decision accuracy and lower physiologically measured cognitive stress, but longer decision times—highlighting the trade-off between accuracy and time sensitivity. In one case, a potentially unsafe recommendation emphasized the need for human oversight and robust safety safeguards [37].

These observations are directly relevant to our findings. Our results show that the largest performance gains in the short-interval training group concerned non-technical and process-related items — structured assessment of reversible causes, team coordination, and timely transitions — precisely the dimensions that current device-based feedback systems cannot capture but that AI-assisted video or audio analysis could plausibly evaluate at scale. A checklist such as the one validated in this study could serve as a structured reference standard against which future AI-supported supervision tools are trained and benchmarked, linking objective process-quality assessment to automated feedback in both simulation-based education and real clinical resuscitation.

This study, therefore, anticipates a shift from purely mechanical CPR optimization to comprehensive management and team performance monitoring. Low-dose, high-frequency training offers one feasible avenue for integrating continuous competence development into daily emergency medical practice. Future research should investigate whether AI-assisted supervision tools translate into improved patient outcomes and clarify their role in augmenting, rather than replacing, human clinical expertise.

### Strengths and limitations

Strengths of this study include its randomized design, one-year follow-up, validated assessment tool with good inter-rater reliability, and external blinded validation. The focus on management and non-technical skills represents a relevant extension of existing CPR research.

Limitations include the simulation-based design without patient-centered endpoints, the relatively small sample size, lack of participant blinding, and potential Hawthorne effects. Although the self-developed rating instrument has been validated, it is not yet internationally established. In addition, the trial involved multiple comparisons across the primary outcome, domain-specific scores, time-to-intervention measures, and questionnaire items, and no formal adjustment for multiple testing was applied. The risk of Type I error may therefore be inflated, and individual statistically significant findings should be interpreted with caution.

Several aspects further constrain the generalizability of our findings. First, performance in a high-fidelity simulation does not necessarily translate to performance during real out-of-hospital cardiac arrest, where stress, environmental constraints, and patient variability differ substantially. Second, the trial was conducted exclusively in an out-of-hospital adult resuscitation context; transferability to in-hospital cardiac arrest, where team composition, equipment, and response dynamics differ, cannot be assumed. Third, the study population consisted of adult OHCA scenarios only; the findings should not be extrapolated to pediatric or neonatal resuscitation, which involves distinct algorithms, team structures, and cognitive demands. Fourth, the trial was conducted within a single German EMS system with a specific tiered response model and provider qualification structure; results may differ in EMS systems with other staffing models, training traditions, or scope of practice. Finally, participants enrolled voluntarily and may have been more motivated than the general EMS workforce; the effect of short-interval training on a non-volunteer or mandated cohort remains to be investigated. Multicenter studies with patient-centered outcomes and health-economic analyses are warranted.

## Conclusion

In this randomized, simulation-based trial, low-dose, high-frequency ALS training resulted in higher resuscitation performance scores after twelve months than annual full-day refresher training. The largest between-group differences were observed in non-technical and process-related items, while basic mechanical CPR parameters did not differ meaningfully between groups. Whether these simulation-based findings translate into improved performance in real cardiac arrest care remains to be determined.

## Supplementary Information

Supplementary Material 1.

Supplementary Material 2.

## Data Availability

Data are available from the corresponding author upon reasonable request and with permission of the respective research institution.
