# Supplementary material for: Effect of low-dose, high-frequency advanced life support training versus annual full-day training on simulation-based resuscitation performance: a randomized controlled trial
Source: BMC Med Educ. 2026 Jun 20;26:1014. doi: 10.1186/s12909-026-09717-3 (PMC13285054; doi:10.1186/s12909-026-09717-3)
Supplement: Supplementary file 1 — Supplementary Material 1. [file 12909_2026_9717_MOESM1_ESM.pdf]

participant survey

Dear colleagues,
As part of my doctoral thesis and study *entitled "Impact of short interval training on the quality of simulation-based cardio-pulmonary resuscitation. A prospective randomized control trial (Effect-CPR),"* I would like to ask you to complete the following questionnaire:

Last digit of your year of birth:	
First letter of mother's first name:	
First letter of your father's first name:	
First letter of your place of birth:	

1. Gender: ☐ Female ☐ male ☐ diverse

2. Age: .... in years

3. Qualification : ☐ Emergency medical technician ☐ Paramedic ☐ Emergency medical technician ☐ Emergency paramedic ☐ Nurse ☐ Specialist nurse - ☐ Physician ☐ Other:................ Further training

4. completed Years of service in active emergency medical services/nursing: ....

5. have you participated in resuscitation training?
   Yes ☐ No ☐

6. What type of resuscitation training have you participated in?
   ☐ ALS course ☐ BLS course ☐ University studies ☐ Emergency medical services ☐at work
   ☐ Other: ....

7. How often do you participate in CPR training?
   ☐ Monthly ☐ Every 3 months ☐ every 6 months ☐ Once a year
   ☐> once a year

8. When was the patient last resuscitated?
   ☐ 4 weeks ago ☐ 3 months ☐ 6 months ☐ 1 year ☐ >1 year

translated questionnaire

EBERHARD KARLS UNIVERSITÄT TÜBINGEN

Universitätsklinikum Tübingen

participant survey

	1 (fully applicable)	2	3	4 (not applicable)
I feel confident in guiding my team during CPR				
Do you feel adequately trained in the procedure and algorithm for CPR?				
Do you feel experienced enough to maintain/guide a CPR cycle in accordance with guidelines?				
Would you feel confident in assessing the different ECG rhythms?				
Would you be confident in assessing the various ECG rhythms?				
Would you feel confident in correctly treating the different ECG rhythms?				
Do you feel adequately trained in administering medications during resuscitation?				
Are you adequately trained in administering medications during resuscitation?				
Are you familiar with all the potentially reversible causes of cardiac arrest?				
Can you recognize ROSC during resuscitation using specific parameters?				
Are you familiar with the ROSC algorithm?				

translated questionnaire
