# Supplementary material for: Effect of low-dose, high-frequency advanced life support training versus annual full-day training on simulation-based resuscitation performance: a randomized controlled trial
Source: BMC Med Educ. 2026 Jun 20;26:1014. doi: 10.1186/s12909-026-09717-3 (PMC13285054; doi:10.1186/s12909-026-09717-3)
Supplement: Supplementary file 2 — Supplementary Material 2. [file 12909_2026_9717_MOESM2_ESM.pdf]

Effect-CPR – Checklist

Video:

	Rating	5	4	3	2	1
	**Initial Approach**					
1.	Cardiac arrest recognized promptly	☐	☐	☐	☐	☐
2.	Immediate start of CPR after recognition of cardiac arrest	☐	☐	☐	☐	☐
3.	High-quality chest compressions (depth, rate, minimal interruptions, etc.)	☐	☐	☐	☐	☐
4.	Adequate ventilation (respiratory rate, tidal volume)	☐	☐	☐	☐	☐
5.	Airway management, 100% oxygen, prompt capnography	☐	☐	☐	☐	☐
6.	Prompt IV access	☐	☐	☐	☐	☐
	**Shockable rhythm**					
7.	Rhythm analysis, defibrillator charged promptly during CPR	☐	☐	☐	☐	☐
8.	Rhythm correctly interpreted	☐	☐	☐	☐	☐
9.	Defibrillation with adequate energy and safety precautions	☐	☐	☐	☐	☐
10.	2 minutes of CPR (compressions: depth, rate, recoil, minimal interruptions; ventilation: rate, tidal volume)	☐	☐	☐	☐	☐
	**Shockable rhythm**					
11.	Rhythm analysis, defibrillator charged promptly during CPR	☐	☐	☐	☐	☐
12.	Rhythm correctly interpreted	☐	☐	☐	☐	☐
13.	Defibrillation with adequate energy and safety precautions	☐	☐	☐	☐	☐
14.	2 minutes of CPR (compressions: depth, rate, recoil, minimal interruptions; ventilation: rate, tidal volume)	☐	☐	☐	☐	☐
	**Non-shockable rhythm**					
15.	Rhythm analysis, defibrillator charged promptly during CPR	☐	☐	☐	☐	☐
16.	Rhythm correctly interpreted	☐	☐	☐	☐	☐
17.	2 minutes of CPR (compressions: depth, rate, recoil, minimal interruptions; ventilation: rate, tidal volume)	☐	☐	☐	☐	☐
	**Non-shockable rhythm**					
18.	Rhythm analysis, defibrillator charged promptly during CPR	☐	☐	☐	☐	☐
19.	Rhythm correctly interpreted	☐	☐	☐	☐	☐
20.	2 minutes of CPR (compressions: depth, rate, recoil, minimal interruptions; ventilation: rate, tidal volume)	☐	☐	☐	☐	☐
	**General**					
21.	Prompt administration of correct medications and dosing	☐	☐	☐	☐	☐
22.	Reversible causes (Hs and Ts) considered and treated as needed	☐	☐	☐	☐	☐
23.	Medication administered promptly with every second rhythm analysis	☐	☐	☐	☐	☐
24.	Compressor change every 2 minutes	☐	☐	☐	☐	☐
25.	Team avoids unnecessary actions at inopportune times	☐	☐	☐	☐	☐
26.	<u>Situational awareness and decision making</u><br>The team anticipates and reassesses the situation, monitors the workflow, and adapts to changing circumstances.	☐	☐	☐	☐	☐
27.	<u>Leadership and teamwork</u><br>The team leader visibly leads the team and coordinates teamwork. Team members follow the leader and complete the task together.	☐	☐	☐	☐	☐
28.	<u>Workload distribution</u><br>The team sets priorities dynamically, coordinates activities, and follows standards.	☐	☐	☐	☐	☐
29.	<u>Communication</u><br>The team communicates effectively, anticipates, and shares information. Plans and intentions are clearly stated.	☐	☐	☐	☐	☐
